# Predicting the Uncertain Future of Aptamer-Based Diagnostics and Therapeutics

**DOI:** 10.3390/molecules20046866

**Published:** 2015-04-16

**Authors:** John G. Bruno

**Affiliations:** Operational Technologies Corporation, 4100 NW Loop 410, Suite 230, San Antonio, TX 78229, USA; E-Mail: john.bruno@otcorp.com; Tel.: 1-210-731-0015 (ext. 2228); Fax: 1-210-731-0041

**Keywords:** antibody, aptamer, diagnostic, humanized, modified base, multivalent, therapeutic

## Abstract

Despite the great promise of nucleic acid aptamers in the areas of diagnostics and therapeutics for their facile *in vitro* development, lack of immunogenicity and other desirable properties, few truly successful aptamer-based products exist in the clinical or other markets. Core reasons for these commercial deficiencies probably stem from industrial commitment to antibodies including a huge financial investment in humanized monoclonal antibodies and a general ignorance about aptamers and their performance among the research and development community. Given the early failures of some strong commercial efforts to gain government approval and bring aptamer-based products to market, it may seem that aptamers are doomed to take a backseat to antibodies forever. However, the key advantages of aptamers over antibodies coupled with niche market needs that only aptamers can fill and more recent published data still point to a bright commercial future for aptamers in areas such as infectious disease and cancer diagnostics and therapeutics. As more researchers and entrepreneurs become familiar with aptamers, it seems inevitable that aptamers will at least be considered for expanded roles in diagnostics and therapeutics. This review also examines new aptamer modifications and attempts to predict new aptamer applications that could revolutionize biomedical technology in the future and lead to marketed products.

## 1. Introduction

Correctly predicting the future of aptamers is not an easy task. Despite the great promise of aptamers to possibly replace or at least effectively compete with antibodies in diagnostics and therapeutics for the advantages presented in Table 1, aptamer commercialization has not yet come to fruition. Aptamers have not substantially supplanted antibodies in either diagnostics or therapeutics after nearly 25 years of development, yet aptamers are not a complete failure either. Despite the paucity of commercial success with aptamers or aptamer-based products, aptamers continue to be a fervent topic of research, because they perform well and hold the advantages listed in Table 1. This fervor is supported by a perusal of PubMed using “aptamer” as a search term, which currently returns nearly 4900 articles, so before critics condemn aptamers to the garbage heap of scientific history, it would be wise to reconsider the promise and clear advantages of aptamers. Monoclonal antibody technology required approximately four decades to come to fruition at great expense to biotechnology and pharmaceutical companies which needed to humanize the antibodies. It is that huge investment in antibody technology which may also be partly responsible for the resistance of major companies toward aptamer technology.

The demise of Archemix, Inc. (Cambridge, MA, USA) several years ago and the halting of Phase 3 clinical trial enrollment for Regado Bioscience’s Revolixys kit composed of pegnivacogin, an anticoagulant aptamer against Factor IX, and its complementary oligonucleotide, were significant setbacks to therapeutic aptamer ambitions. These failures leave Macugen^®^ as the only FDA approved and marketed therapeutic aptamer. The early struggles of aptamer therapeutics coupled with the huge investment of pharmaceutical companies in humanized monoclonal antibodies and other biologics do not bode well for aptamer therapeutics. However, there are some exciting new means to protect and enhance aptamers *in vivo* as well as new therapeutic approaches and applications described in this article which could positively alter the track record of aptamers.

Only a handful of companies have thus far developed and marketed aptamers as antibody-like binding reagents for research and development including the author’s company (OTC Biotech, San Antonio, TX, USA), Aptagen (Jacobus, PA, USA), Base Pair Biotechnologies (Houston, TX, USA), NeoVentures Biotechnologies (London, ON, Canada), Aptamer Sciences (Pohang, Korea) and a few others. Even fewer companies have marketed aptamers as components of assay kits or concentrating devices, but among these are the NeoVentures Ochratoxin A and aflatoxin ELISA-like microplate assays [1] and affinity columns. The seminal aptamer company SomaLogic (Boulder, CO, USA) appears to be pushing its SOMAmers and SOMAScan aptamer array platform sensor into the proteomics and diagnostics markets via beta testing at various locations with a current capability to detect greater than 1100 different human proteins to sub-pg levels in body fluids [2,3].

The fact that aptamers first emerged on the biotechnology landscape in the early 1990s and have had meager commercial success should not dissuade researchers from continuing to pursue aptamer use and innovation. In the author’s own experience, one of his company’s C-Reactive Protein (C-RP) aptamers led Vance and Sandros [4] to develop a nanoparticle-based Surface Plasmon Resonance (SPR) assay with low zeptomolar (10^−21^ M) detection of C-RP even when conducted in human serum. And in another of the author’s own research efforts, he was able to develop a Brain Natriuretic Peptide (BNP) aptamer sandwich assay using electrochemiluminescence (ECL) in serum with sub-pg/mL sensitivity [5]. These and other recent successes in specific detection of analytes at ultrasensitive levels should encourage researchers to continue the pursuit of aptamer-based assays and therapeutic technologies. This review provides a vision and partial roadmap for where aptamers and their applications may progress. The author wrote a review of aptamer conjugates in 2013 [6] which described in some detail the state of the art for aptamer conjugates, so to avoid redundancy, mostly new areas or updates to previously discussed aptamer and aptamer conjugate technologies are addressed herein. Other authors have pointed out *potential* advantages of aptamers such as potentially greater affinity or specificity *versus* antibodies for some aptamers developed under stringent conditions. However, Table 1 lists unequivocal advantages of all aptamers *versus* antibodies which could lead to niche markets for aptamers.

**Table 1:** Key Advantages of Aptamers *vs.* Antibodies with Supportive References.

Advantage	References
Facile *in vitro* development which obviates host animals.	[7]
Ability to develop aptamers against native toxins without toxoid production.	[7]
Greater reproducibility of aptamers from batch-to-batch due to chemical synthesis	[7]
More rapid ability to develop neutralizing agents by robotic means against multi-drug resistant “doomsday bug” bacteria or emerging lethal viruses (e.g., Ebola, influenzas, MERS, SARS, *etc.*) for prophylaxis or a “bridge to life” prior to host-mounted immune response (*i.e.*, prior to seroconversion).	[8,9,10,11,12]
Ability to develop aptamers onboard spacecraft, other planets, or in remote locations on Earth where neutralizing aptamers may be needed.	[13,14,15]
Unlimited inexpensive production of DNA aptamers at the gram or greater scale by PCR or asymmetric PCR (predominately single-stranded PCR products).	[16,17]
Ability to store lyophilized aptamers indefinitely and obviate cold storage.	[18]
Reusability; aptamers can be heat-denatured, cooled to reconform and used for many rounds of analyte binding and detection.	[19,20,21]
Little or no immunogenicity.* Even humanized mAbs can be immunogenic.	[22,23,24]

* The work of Wendel’s group [23] suggesting immune system activation by some aptamers and the need for pre-clinical testing are duly noted.

## 2. New Diagnostic Aptamer Reagent Innovations and Marketing Strategies

There is always room for new diagnostic modalities and assay formats, such as aptamer-nanoparticle conjugate surface-enhanced Raman spectroscopy (SERS) [25] or aptamer-coated magnetic beads to concentrate and purify analytes from body fluids prior to mass spectral analyses [26]. Coupling either SERS or liquid chromatography (LC)-mass spectral analyses of body fluids to aptamer technology could be huge and lucrative applications in various parts of the diagnostics industry. However, the dominant trend in recent aptamer-based diagnostics appears to relate to the composition of aptamers. This focus is represented by the subcategories below discussing longer multivalent aptamers and the addition of unnatural bases or alterations to the backbone and sugars to potentially enhance affinity, avidity, specificity and stability of aptamers.

Additionally, researchers appear to be focusing on the so called “low lying fruit” in diagnostics, namely food safety and environmental assays or “home brewed” laboratory-developed tests (LDTs) for clinical analytes [27]. LDTs are less expensive tests for clinical analytes used within the confines of a single laboratory or company. Although currently under increased scrutiny by the FDA, LDTs could provide a short-term “back door” route for commercialization of some aptamer-based clinical assays, if an entrepreneur was so inclined. The FDA will phase in LDT regulations over the next 9 years [28,29]. If one considers the huge success of Elizabeth Holmes’ recent $9 billion valuation for Theranos, Inc. (Palo Alto, CA, USA), which is brilliantly based in part on taking advantage of LDTs, teaming with pharmacies as blood collection and testing centers to lower costs, increased reporting speed and greatly reduced blood draw volumes (a few drops of blood), companies should take LDTs and other aspects of Theranos business plan quite seriously in their strategic plans. Although somewhat controversial for their ability to “side step” FDA regulations, LDTs are considered by some experts to be the future of personalized medicine and are used for detection of rare diseases that might otherwise be ignored for assay development by larger diagnostic companies because such companies see little or no profit in investing in rare disease diagnostics. Of course, supplementing the LDT strategy by simultaneously seeking FDA approval for aptamer-based tests would seem to be the wisest long-term course of action.

### 2.1. Longer Multivalent or Multidentate Aptamers

At the outset of this section, it is important to state that the terms “multivalent” and “multidentate” are often used interchangeably with regard to aptamers. Early usage of the term multivalent by Shi and Lis [30] meant artificial composite or concatenated aptamers which were engineered. However, it is very important to distinguish between intelligently designed multiple binding site aptamers and those which are naturally selected by providing longer randomized DNA template starting material. Ahmad *et al.* [31] have demonstrated that nature is still the supreme engineer, because it is quite difficult for any human to precisely know and engineer a multiple binding site aptamer with perfect linker spacing and flexibility to fit a complex antigen’s topology [32]. Of course, the author acknowledges that there are rare cases in which one can engineer effective bidentate or multidentate aptamers, if detailed knowledge of proximal epitopes and their spacing on complex targets is available, but in general, natural selection will probably produce superior affinity or avidity and specificity.

In the past, commercially available DNA oligonucleotide aptamer length was limited to ~70 bases due to the low yields of lengthier aptamer templates by chemical synthesis. However, recent advances in chemical synthesis have enabled aptamer template lengths up to 200 bases (e.g., Ultramers^®^ by Integrated DNA Technologies, Inc. Coralville, IA, USA). Longer templates mean that the degenerate (randomized) mid-region can be lengthened to better mimic the 110 amino acid complementarity determining regions (CDR) of antibodies which harbor three hypervariable antigen binding regions per CDR per heavy or light chain (12 hypervariable binding sites per antibody molecule) [6]. By analogy, the author’s group has lengthened the degenerate region of their aptamer DNA template to 164 randomized bases with flanking 18 base constant primer regions. So, logically more binding regions may emerge with longer aptamers. The author has seen numerous complex loop structures in these longer multivalent aptamers which probably confer greater specificity for complex multi-epitope targets such as cell surfaces [6,33].

**Figure 1:**
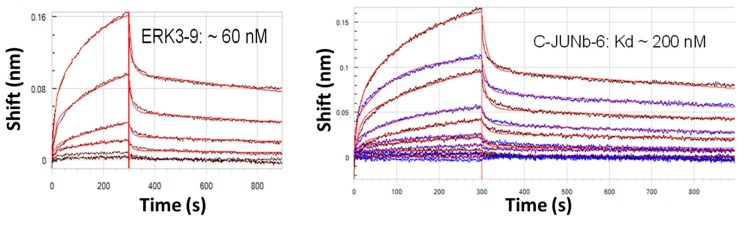
Inverted “convex” interferometer scans of 200 base DNA aptamers binding small cancer biomarker peptides. The unprocessed curves (not shown) are “concave” due to blue-shifting of the smaller bound aptamer-peptide complex as compared to the larger free aptamer without its captured peptide. This phenomenon strongly suggests an induced fit binding mechanism with longer aptamers.

Another interesting aspect that has emerged from working with longer 200 base aptamers is the observation of induced fit around a small target molecule. In recent work, the author’s group developed ~200 base DNA aptamers against several cancer biomarker peptides. When these aptamers were analyzed to determine their affinity constants by interferometry using a Pall Corp./Forte Bio Octet^®^ instrument, the analysis produced blue-shifted instead of red-shifted binding data suggesting that the aptamers actually contract and became smaller upon binding the peptide. This event may be thought of much like a flower closing its petals around the flower’s ovary and other core structures or a Venus flytrap snapping shut around a captured fly upon landing (*i.e.*, a form of induced fit). The blue-shifted spectra appear like a concave dissociation curve (not shown). Figure 1 shows the binding curves of two 200 base aptamers binding their cognate cancer biomarker peptides (K_d_ values of 60 and 200 nM) after they have been inverted back to a more conventional and recognizable “convex” binding curve similar to traditional red-shifted interferometry association curves.

### 2.2. The Use of Exotic, Modified or Unnatural Bases

**Figure 2:**
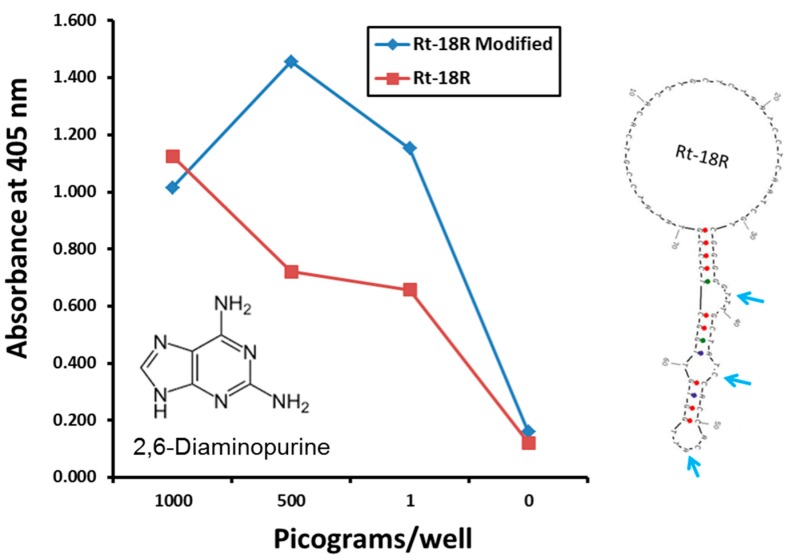
Improved *Rickettsia* aptamer performance in an ELISA-like assay with diaminopurine (DAP) built into three putative *R. typhi* aptamer binding pockets. *Rickettsia* cells were measured by weight since absolute cell numbers were not known or provided by the source to the author’s laboratory.

Criticism of aptamers has always largely centered on the limited monomer repertoire of a mere four nucleotides *versus* the diverse 20 amino acids available to antibodies, but this is changing [34,35]. The availability of exotic and unnatural base incorporation from DNA synthesis vendors for rationally engineered placement in putative binding pockets (e.g., Figure 2) is proving advantageous for improved assay performance. In the author’s laboratory, the addition of diaminopurine bases into the middle of three putative *Rickettsia typhi* aptamer pockets (secondary loop structures) appeared to bolster detection of the rickettsia in the low pg range (Figure 2). This is a simple example of enhanced performance of course, but it illustrates the principle of post-selection molecular engineering of aptamers for improved affinity. The ability to build disulfide bonds into aptamer bases [7] could also, in theory, be used to stabilize critical tertiary aptamer structures that might otherwise change with temperature, pH, salt concentration or other ionic conditions.

Random placement of unnatural or modified bases using more promiscuous DNA polymerases without editing functions is also an option, but then standard sequencing is impossible and the selected aptamer must be sequenced by exonuclease digestion and mass spectral analyses [36]. AM Biotechnologies, Inc. (Galveston, TX, USA) is marketing an “X-aptamer” kit [37] that will yield modified base aptamers, but these must be returned to the company for sequence determination and synthesis. Someday, nanopore sequencing may be able to routinely identify the positions of unnatural or modified bases in aptamer chains.

Perhaps the most radical new development in the area of modified base aptamers is that of “Seligos” produced by Apta Biosciences, Ltd. of Singapore and the UK (a recent spinoff of Fujitsu Laboratories). These unique constructs consist of a DNA scaffold with pendant amino acids to emulate peptides or proteins for binding to target molecules [38].

### 2.3. Modifications to Aptamer Backbone and Sugar Moieties

Aptamer technology has long relied on several modifications to the phosphate backbones and sugar moieties of DNA and RNA to enhance aptamer stability *in vivo*. These modifications have included substitution of sulfurs for non-bridging oxygens in the phosphate backbone (*i.e.*, phosphorothioate or thioaptamers) which retard degradation in serum while also enhancing binding affinities [7,39]. Other staple means to protect aptamers from serum endo- or exonucleases have included modification of deoxyribose at the 2' position with amine, fluoro, methoxy, and thiol groups [7,39].

Recently, the emergence or “synthetic genetic polymers” or “xeno nucleic acids” (XNAs) has offered several more options for unnatural polymers that function like DNA and RNA for the passage of genetic information [40,41,42]. However, XNA backbones should be poorly recognized by natural nucleases and thus should be highly nuclease resistant and exhibit longer half-lives *in vivo* [42]. Novel XNAs include polymers composed of modified sugars such as those in arabinonucleic acids (ANA), cyclohexenyl nucleic acids (CeNA), 2'-fluoroarabinonucleic acids (FANA), 1,5-anhydrohexitol nucleic acids (HNA), locked nucleic acids (LNA), peptide nucleic acids (PNA) and α-L-threofuranosyl nucleic acids (TNA) [40]. These XNAs can be used to develop aptamers by classic methods, if polymerases exist which are able to amplify them following a round of affinity selection. Fortunately, variants of the *Thermococcus gorgonarius* polymerase (e.g., TgoT) can be evolved to amplify various XNAs [40], thus providing real hope that this method may produce ultra-stable aptamers for use *in vivo*. Of course, the downside to using any non-DNA or non-RNA aptamer is still the potential for very adverse immune reactions especially upon subsequent administration to patients.

### 2.4. One Step and In Silico Aptamer Development

Researchers continue to streamline and refine the classic Systematic Evolution of Ligands (SELEX) aptamer development process [43,44,45,46], chasing the holy grail of reliable one step aptamer selection [46] instead of the repetitive select and amplify cycles followed by cloning and sequencing. Unfortunately no simplified or expedited SELEX process has yet emerged to challenge the gold standard Slow Off-rate Modified Aptamers (SOMAmers) [2,3]. But, this is clearly an area of potential future improvement, so it should be included in this vision of future aptamer technology. One potential way to achieve one step aptamer identification may be *in silico* computer modeling. Great strides are being made in this area with new 3-D docking [47], free energy and entropy-based “seed and grow” algorithms [48,49,50,51,52]. As a veteran of many rather laborious aptamer “fishing trips,” the author dreams of either robotic or *in silico* solutions to aptamer development. The only drawback to *in silico* aptamer development is that one must know the exact composition and topology of the target (unknown targets are of course excluded from the *in silico* approach) and the more complex the target is at the molecular level, the more intense the computations. Still, *in silico* aptamer development is clearly a promising area to watch, because it could dominate aptamer diagnostic and therapeutic development in the future.

### 2.5. Environmental, Food Safety and Other Niche Market Aptamer Assays

A number of groups, including the author’s group, have tried to push aptamers into the environmental niche markets (including chemical and bioterrorism assays [53,54,55,56,57]) and food safety testing [58] with some very sensitive and specific assays for the major foodborne bacterial pathogens [59,60,61,62,63,64,65,66,67,68,69,70,71], troublesome noroviruses [72,73,74], foodborne toxins [1,75], antibiotics [76,77] and pesticides [25,78,79]. At first glance, it seems that market entry in food safety testing should be relatively facile because the developer only needs to pass AOAC certification to begin selling kits, assays or instruments, but the USDA and FDA have a zero tolerance policy for any foodborne pathogens in large volumes of foods. Hence, food safety testing becomes an effort to develop extremely high sensitivity and rapid assays in order to probe large volumes in minimal time. Ultrasensitive aptamer-coated magnetic bead concentrating methods to probe foods for rare pathogenic cells or viruses coupled to PCR detection assays [80,81,82] or fluorescence detection [59,64] have been shown to cut hours, if not days, off of culture enrichment and reporting times. In addition, the author recently published the first aptamer-quantum dot lateral flow test strip assay report for highly sensitive detection of several foodborne pathogens with sensitivities approximately ten-fold greater than antibody colloidal gold lateral flow assays [66]. Yet, no one has marketed an AOAC-approved aptamer-based foodborne pathogen test to date. This may be due to general ignorance of aptamers in the food safety testing world and perhaps success in this arena is just around the corner. Some success has already been realized by NeoVentures Biotechnologies, Inc. (London, ON, Canada) which markets aptamer-based concentrating and purifying columns and assay kits for ochratoxin A and aflatoxins in corn, wheat, beer and wine [1].

## 3. New Therapeutic Innovations and Applications

Aptamer therapeutics advocates have little to laud except the late 2004 FDA approval of Macugen^®^ (pegaptanib) which has held its own in the market against the monoclonal antibodies Avastin^®^ (bevacizumab) and Lucentis^®^ (ranibizumab) for the treatment of wet age-related macular degeneration. However, in the latest 2014 study of 3180 reports of adverse drug events in a WHO database of patients administered these three VEGF antagonistic drugs, Avastin^®^ appeared to be the winner for its “favorable safety profile and lower cost” [83]. Regardless of whether Macugen^®^ is the top treatment for macular degeneration or not, it was definitely a moral victory and success story for aptamers in general. Ni *et al.* [84] and Sundaram *et al.* [85] have nicely summarized the remaining therapeutic aptamers which may still be in pharmaceutical development pipelines somewhere. The following sections look to the future for the next potential therapeutic aptamer or aptamer derivative victories in several diverse areas of medicine.

### 3.1. Update on Aptamer Conjugates

The author previously summarized the aptamer-siRNA work of Rossi [6,86] and others as well as the aptamer-alpha gal epitope conjugate work of Altermune, LLC (Irvine, CA, USA; founded by PCR inventor and Nobel prize laureate Kary Mullis) [6]. So, this section consists of updates related to exciting new aptamer-based drug delivery reports, new data and previously unmentioned work since the previous summary [6].

**Figure 3:**
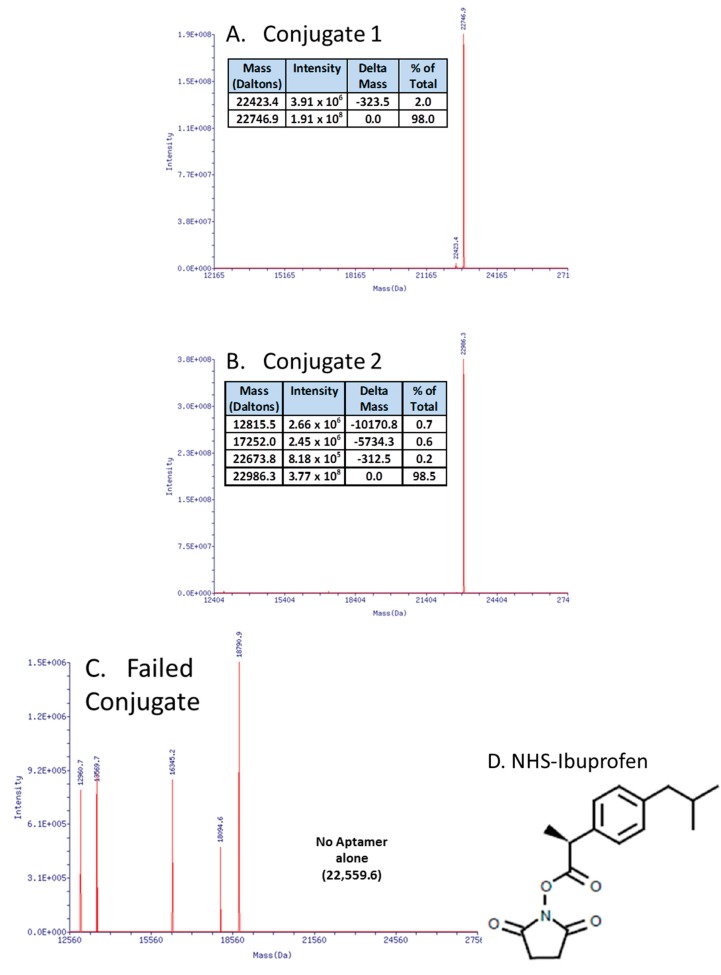
Mass spectral data showing successful aptamer-3'-ibuprofen conjugation (**A**,**B**) *versus* the surprisingly fragmented DNA resulting from a carbodiimide-mediated approach (**C**). The successful approach involved the use of a custom N-hydroxysuccinimide (NHS)-ibuprofen linker synthesized by Sigma-Aldrich Corp. (**D**) resulting in >98% yields of the correct conjugates (actual *vs.* theoretical molecular weights matched for aptamer-ibuprofen conjugates as determined by mass spectra in panels A and B).

In recent work (Figure 3A,B), the author essentially replicated the work of Manoharan *et al.* [87] who coupled ibuprofen to an antisense oligonucleotide in order to “hitch a ride” on albumin and other serum proteins, thereby enhancing an antisense oligonucleotide’s serum half-life. This strategy of attaching ibuprofen or other NSAIDs to DNA (instead of polyethylene glycol (PEG) or other molecular “anchors”), especially on the 3' end to affiliate with serum proteins and thus increase the aggregate complex’s weight, thereby slowing kidney and liver clearance and inhibiting exonuclease degradation [88,89,90] may be useful for aptamers as well. The surprising aspect for the author was that carbodiimide (EDC or EDAC)-based coupling of aptamer-3'-primary amines with the carboxyl group of ibuprofen in MES buffer at pH 5.0 failed repeatedly with strong evidence of DNA fragmentation and no evidence of successful ibuprofen coupling by mass spectral analyses (e.g., Figure 3C). Instead, a custom-synthesized (Sigma-Aldrich, Saint Louis, MO, USA) N-hydroxysuccinimide (NHS) version of ibuprofen (Figure 3D) led to >98% yield of the correct conjugates as determined by electrospray ionization mass spectral analyses at Integrated DNA Technologies, Inc. (Coralville, IA, USA) (Figure 3A,B). The author is in the process of animal testing of the aptamer-3’-ibuprofen conjugates and expects to report their pharmacokinetics in the next year or two.

### 3.2. Aptamers to Neutralize Lethal Viruses

HIV continues to be a target of fervent aptamer development [91]. However, breach of U.S. borders by Ebola last year, and the emergence of new viral threats in America such as MERS, Dengue, West Nile Virus, and most recently the Bourbon virus, may shift some aptamer development efforts to these newer viral threats [12,92,93,94]. It appears clear that high affinity and highly specific aptamers can bind envelope, polymerase and structural viral proteins to inhibit or block fusion, penetration, or replication of influenza [11,94,95,96,97,98] and perhaps other important viruses [99]. Hence as new viral threats emerge, aptamer research and development seem likely to parallel viral emergence, because in the absence of effective vaccines or antisera, aptamers may provide a last line of defense. Indeed, in the event of another lethal pandemic influenza or other virus outbreak, aptamers may provide the only line of defense and could be developed robotically [8,9,10], if necessary to avoid human exposure to the virus, for passive immunity until patients can mount their own immune responses.

### 3.3. Aptamers or Aptamer Conjugates to Kill Drug-Resistant Pathogenic Bacteria

The crisis of antibiotic resistance which is already upon the human race demands either new antibiotic development or novel approaches to killing multidrug-resistant (MDR) bacteria *in vivo*. Recently, carbapenem-resistant *Enterococcus* (CRE) and *Klebsiella pneumoniae* (KPC) have garnered most of the morbid headlines for nosocomial acquisition and high patient mortality in hospitals while MRSA and VRE continue to be significant problems. Unfortunately, the new drug pipeline for resistant bacteria is scant which has drawn attention to chimeric or humanized monoclonal antibodies to combat drug-resistant bacteria [100,101,102]. Similarly, aptamers can perhaps be used as bacteriostatic agents to possibly inhibit the growth of bacteria by simple cell surface binding to decrease membrane potential [103]. Aptamers are being investigated to determine if they can block or degrade key drug-resistant bacterial enzymes such as New Delhi metallo-β-lactamase (NDM-1), thereby possibly overcoming resistance to beta-lactams and a variety of other antibiotics [104]. Direct killing of Gram negative bacteria and cancer cells by induction of the classic complement-mediated cell lysis system [6,105,106,107] or opsonization [108,109] by bifunctional (bidentate linker) aptamers or aptamer-Fc conjugates to emulate antibodies has also been demonstrated. Hopefully, these approaches will be further investigated and exploited in the future to combat MDR bacteria, cancers [106,107], and even some parasitic infections.

### 3.4. Aptamers to Neutralize Toxins and Venoms

Antibodies must often be made against chemically fixed or heat-inactivated toxins (toxoids) for injection into host animals. But, toxoids do not provide the same tertiary conformations as native toxin proteins for antibody development. This is not a problem for aptamers which are developed *in vitro* against native protein conformations [7]. To date, several highly specific aptamers have been developed against a variety of different bacterial toxins [75,110,111] and snake venoms [112,113]. Some of the nascent aptamers also demonstrated significant (up to 25%) inhibition of sphingomyelinase activity in brown recluse spider venom [114]. Of course, snake and other venoms are complex materials which can include phospholipases and other degradative enzymes as well as neurotoxins and cardiotoxins in some cases. These toxic components would all require neutralization by a cocktail of “polyclonal” aptamers to be effective as a single antivenom product. While stoichiometric aptamer scavengers may not be pragmatic *in vivo* if high doses are required for 1:1 aptamer:toxin stoichiometry, any aptamer which binds a toxin or venom component specifically, could possibly be converted into a DNAzyme or aptazyme with the proper molecular engineering. And enzymatic aptamers would reduce the requisite antidote dose. These thoughts inspire the hope that perhaps aptamers or their conjugates can be developed to effectively counteract snake, spider, scorpion, insect and other venoms. Antisera for venomous bites require cold storage, but lyophilized aptamers would not require cold storage and could be kept at ambient temperatures (even in desert or jungle environments) for long-term storage [18] as long as humidity was sealed out.

### 3.5. Robotic Aptamer Development and Production for Rapid Medical Countermeasures

In 1996, the author was the first scientist to publish the use of target molecules covalently attached to magnetic microbeads to act as affinity targets for SELEX aptamer development [115]. This approach stemmed from frustration with the larger volume ethanol precipitation step which was unreliable in the SELEX process [116]. The author was also the first to develop aptamers against whole bacterial cells in the form of *B. anthracis* spores with centrifugal partitioning of spore-bound and unbound supernate DNA from the aptamer pool [117]. The use of these two techniques enables facile robotic aptamer development as shown in other published works [9,10]. While automated robotic platforms could be quite useful for routine aptamer product development, there are two futuristic, yet plausible, scenarios in which they could be paramount to human survival. These scenarios are: (1) if humanity was faced with a devastating pandemic “doomsday bug” (viral or bacterial), even if the microbe was of completely unknown identity or origin and (2) if astronauts in outer space or on other planets encountered a deadly new pathogen capable of rapidly defeating their immune systems. In both cases, automated robotic SELEX platforms could be immensely valuable countermeasures for allowing sick humans to develop aptamers for passive immunity until their own immune systems could respond to the invading pathogen. NASA scientists have already realized and acknowledged this fact in various publications [13,14]. And with recent advances in microfluidics and MEMS (microelectromechanical systems), automated aptamer selection devices in space or on other planets could be quite compact and lightweight [10,118,119,120,121] to accommodate small numbers of humans.

### 3.6. Aptamers to Potentially Induce Stem Cell Differentiation and Transdifferentiation

One very exciting potential application of aptamers could be to induce differentiation or transdifferentiation of stem cells, especially of bone marrow origin, to avoid the controversy surrounding the use of human embryonic stem cells. Regenerative medicine is beginning to demand larger amounts of stem cells and 100% complete differentiation into end cells. Aptamers have already played a part in the affinity-based capture, isolation, concentration and collection of progenitor cells from various tissues [122,123,124,125], proving that whole cell-selected aptamers can be developed and bind stem cells with high affinity. However, the concept for aptamers to replace growth factors and other ligands or agonists rests on the notion that aptamers can bind key receptors on the surface of stem cells to induce differentiation.

One example of aptamer-promoted differentiation of axon outgrowth already exists in the work of Wang *et al.* [126]. Wang *et al.* demonstrated that RNA aptamers bound the Nogo-66 receptor (NgR) and competed with natural myelin-derived inhibitors of NgR consequently promoting axon outgrowth [126]. While this is a somewhat convoluted route to changing cellular morphology and functionality, it illustrates the point that aptamers can play a key part in cell differentiation pathways.

A more direct route to differentiation induction has been laid by combinatorial antibodies [127,128,129]. Richard Lerner’s laboratory at the Scripps Research Institute (La Jolla, CA, USA) has done pioneering work on the use of combinatorial antibodies to act like G-CSF to transdifferentiate CD34+ bone marrow cells into neural progenitors [128]. And in another project, Lerner’s group reported integrin-binding combinatorial antibodies which transformed human stem cells into dendritic cells [129].

As this review and many other articles have pointed out, aptamers can do virtually everything that antibodies can do, but are more facile to produce than virus-displayed combinatorial or humanized antibodies and are more reproducible [7]. Therefore, it seems only a matter of time before reports of aptamer-mediated stem cell transdifferentiation appear in the literature. And because aptamers may be less expensive to produce and generally nonimmunogenic, they could become the agents of choice to replace more expensive recombinant antibodies or growth factors, thereby accelerating the field of regenerative medicine.

### 3.7. Aptamers to Induce Rapid Blood Clotting

The vast majority of work in the area of blood clotting over many years has centered on developing aptamers as regulatable anticoagulants. More recently, the focus appears to be broadening to include aptamers that actually clot blood as a treatment for hemophilia [130]. While binding and activating key proteins in the clotting cascade with aptamers to treat hemophilia is likely to be a new focus in this area, the author can also envision bidentate or multi-dentate aptamers or aptamer-dendrimer conjugates capable of binding proteins, platelets and blood cells in matrices to form clots locally (*i.e.*, like bricks and mortar). Tan’s group at the University of Florida (Gainesville, FL, USA) has recently published an artificial, yet germane, proof of this concept [131]. By synthesizing anti-thrombin aptamer-lipid conjugates that anchor on target cells, Tan’s group was able to form gel-like clots containing the aptamer-modified cells within 15 s in the presence of thrombin and fibrinogen [131]. Clot-inducing aptamers could be dried onto the surface of gauze pads or sponges and applied to patients topically on wounds or internally. Such technology could be invaluable in the life-threatening battle against diffuse bleeding for civilian and military emergency medicine. And an added benefit may be that application of endonucleases might be able to dissolve the aptamer-linked clots as needed.

### 3.8. Mass Production Methods

While aptamers are inexpensive on the milligram-scale by chemical synthesis, their price jumps to ~$2000 per gram by parallel chemical synthesis [6,22]. So, in the future, look for mass production of aptamers by enzymatic methods such as PCR or asymmetric PCR (predominately single-stranded PCR enabled by adding a 100:1 ratio of one primer over the other). Companies such as Vandalia Research (Huntington, WV, USA) are laying the foundation for less expensive mass production of aptamers and other medicinal oligonucleotides with enzymatic techniques such as PCR [16,17] or cell-based production [30].

## 4. Conclusions

The aims of this review were to examine the current state of aptamers in diagnostics and therapeutics and to predict where aptamer technology might progress in the future. In diagnostics, with a few exceptions [1], aptamers are clearly still struggling against entrenched antibody-based assays and the ignorance of the research and development community about aptamer performance and the advantages of aptamers [7,132]. But, the public image of aptamers could change for the better with advances such as longer and more specific multivalent aptamers (up to 200 bases) or the incorporation of modified and unnatural bases or XNAs [40,41,42] for even greater affinity, avidity, specificity and stability. Indeed, SomaLogic’s SOMAmers and their associated diagnostic array platform (SOMAScan) may already be forging a path for diagnostic and proteomic success of aptamers [2,3].

Likewise, therapeutic aptamers have tasted some limited success in the form of Macugen^®^, but the story of therapeutic aptamer efforts has mostly been fraught with failures in late clinical trials. Still, the niches for aptamers in antitoxin, antivenom, MDR bacterial, viral and cancer treatment provide a strong and continuing impetus for eager researchers to keep trying. Thus, it seems a safe bet that aptamers will remain a hot topic of research and development for some years to come. And it is a certainty that such research and development will be quite interesting.
